# Formerly Smoking and Currently Smoking Cancer Survivors’ View on Smoking Cessation – A Qualitative Study

**DOI:** 10.1177/1179173X251355531

**Published:** 2025-10-09

**Authors:** Frederike Bokemeyer, Johanna Springorum, Lisa Lebherz, Carsten Bokemeyer, Holger Schulz, Kathleen Gali, Christiane Bleich, Paulina Kiefer, Sven Püffel, Janina Freitag

**Affiliations:** 1 Department of Medical Psychology, University Cancer Center Hamburg, University Medical Center Hamburg Eppendorf, Hamburg, Germany; 2 Department of Oncology, Hematology, BMT Plus Section Pneumology, University Cancer Center Hamburg, 37734 University Medical Center Hamburg-Eppendorf, Hamburg, Germany; 3 Fresenius University of Applied Sciences, Hamburg, Germany; 4 Hamburg Center for Health Economics, 14915 University of Hamburg, Hamburg, Germany; 5 Cancer Epidemiology and Prevention Group, University Cancer Center Hamburg (UCCH), 37734 University Medical Center Hamburg-Eppendorf(UKE), Hamburg, Germany

**Keywords:** cancer, smoking cessation, nicotine, smoking relapse, psycho-oncology, health service research, qualitative research

## Abstract

**Background:**

Drastic life events, such as a cancer diagnosis, do not necessarily lead to a reduction in unhealthy and dysfunctional behaviors like smoking. Continued smoking among cancer survivors significantly increases the risk of recurrence and worsens treatment outcomes. While evidence-based smoking cessation treatments have demonstrated their effectiveness in acute cancer care, their impact among long-term cancer survivors remains limited, and overall quit rates remain low. To cessation outcome and improve long-term support strategies, it is essential to better understand the experiences, attitudes, and perceived barriers of both current and former smoking cancer survivors.

**Materials and Methods:**

This qualitative study included semi-structured interviews with six cancer survivors (50% female), aged 34 to 81 years, with different cancer types (breast, skin, lung, urinary bladder cancer, and GIST). At the time of the interview, three participants were still smoking, two had quit at the time of their diagnosis, and one had quit beforehand. All had completed cancer treatment at least four years prior without relapse. Interviews were transcribed verbatim and analyzed using qualitative content analysis, applying an inductive approach to identify recurring themes and categorize the data using computer-assisted analysis software.

**Results:**

Four main categories emerged from the interviews: (1) motivations for quitting, (2) perceived barriers, (3) facilitators of cessation, and (4) contextual influences.

Key motives for continued smoking after a cancer diagnosis included managing nicotine cravings and stress, experiencing pleasure and boredom relief, and a perceived lack of negative health consequences. Some participants reported smoking to cope with treatment-related discomfort. A general lack of knowledge regarding the link between smoking and cancer contributed to low motivation to quit and limited risk awareness. Participants who had successfully quit cited improvements in health and well-being as primary reasons for cessation, alongside external factors such as financial savings and the aversion to cigarette odor. Medical advice and support from healthcare providers were mentioned frequently – both as motivating factors and, in cases where such support was absent or discouraging, as barriers.

Barriers to quitting included a persistent smoking environment, negative emotions during cessation attempts, poor timing related to the cancer experience, and previous failed quit attempts.

Conversely, facilitators of cessation included external regulations (eg, smoking bans, legal restrictions), a tobacco-free environment, strong internal motivation, individual coping strategies for withdrawal symptoms and craving, medical recommendations, and support from family or peers.

**Discussion/Conclusion:**

The results highlight the need for a better adaptation of smoking cessation interventions to the specific needs of cancer survivors. This has significant implications for oncology professionals and healthcare providers in cancer care. The inconsistency in smoking cessation advice from healthcare providers, ranging from discouragement to strong encouragement, points to the necessity of re-evaluating current policies and establishing more standardized communication within oncology settings. Cancer survivors require improved education about the health risks associated with continued smoking, as well as information about available cessation aids and pharmacological support options. The “teachable moment” following a cancer diagnosis presents an opportunity to integrate smoking cessation support into routine oncological care. Practical strategies for cancer survivors who wish to quit include the development of alternative behaviors, effective stress management techniques, and further enhancement of legal restrictions to promote a smoke-free environment. Such measures would not only support individual cessation efforts but also contribute to broader public health goals, protecting cancer survivors and the general population from smoking-related harm. The insights from this study provide a foundation for the development of more tailored cessation interventions for cancer survivors.

## Introduction

According to the World Health Organization, smoking is one of the greatest health risks for life-threatening diseases like cancer.^1^ Globally, the number of deaths associated with smoking per year is higher than 8 million.^2^ Numerous studies have documented negative health consequences of continued smoking on both physiological and psychological levels.^3-6^

In patients with cancer, continued smoking is associated with poorer long-term survival, greater risk of treatment complications, and an increased likelihood of developing a second primary cancer or other smoking-related diseases, such as cardiovascular or pulmonary conditions. Furthermore, patients with cancer who smoke often report a reduced quality of life.^4^ As such, the pathway out of smoking addiction is particularly crucial for this vulnerable population, but can be especially challenging.^7^ Leading health authorities, such as the American Association for Cancer Research, the American Society of Clinical Oncology, and the National Comprehensive Cancer Network,^8-11^ advocate for the integration of evidence-based tobacco cessation interventions into standard cancer care. While growing research supports the effectiveness of evidence-based cessation interventions, such as the combination of pharmacological support and behavioral interventions, for cancer survivors, particularly when initiated soon after diagnosis,^12,13^ findings remain mixed. Other studies suggest that existing interventions show limited efficacy among long-term survivors or when more time has elapsed since diagnosis.^14^ In addition, systematic reviews highlight the generally low methodological quality of research in this field.^13,14^

Despite increasing awareness of the harmful consequences of continued smoking, up to 60% of patients with cancer continue smoking after diagnosis.^14,15^ This underscores the need to better understand the experiences, attitudes, and perceived barriers to cessation among cancer survivors in order to optimize and tailor existing interventions. This study aims to address this gap by exploring the perspectives of cancer survivors who currently smoke or have quit smoking.

### Research Questions

The aim of the present research is to explore the motivations, attitudes, and barriers to smoking cessation from the perspective of both former and current smoking cancer survivors. Existing research indicates that smoking cessation interventions are often ineffective for long-term cancer survivors. In this study, we included individuals who had successfully completed their cancer treatment and were declared cancer-free, yet continue to live with the long-term consequences of their illness. In this study these individuals are defined as cancer survivors. They may still face ongoing physical, emotional, or psychological challenges related to their past cancer experience,^16^ which can influence their health behaviors, including smoking.^17^ The findings of this study will provide valuable insights into the experiences of cancer survivors and can contribute to the adaptation of cessation treatments to address the specific needs and concerns of long-term cancer survivors, ultimately improving quit rates and long-term health outcomes.

The following questions were addressed:

1. Which factors motivate cancer survivors to continue smoking?
2. Which factors motivate cancer survivors to cease smoking?
3. Which strategies are used by cancer survivors who have tried or succeeded in quitting smoking?
4. What obstacles are being encountered in the attempt to quit smoking?

## Materials and Methods

### Design

Semi-structured interviews were conducted with cancer survivors who either reported currently or formally smoking. An interview guideline was developed based on smoking cessation literature on the general smoking population^18-23^ and cancer-related smoking population,^24-29^ as well as knowledge that was gained through exchange with firstly smoking cessation experts as and secondly oncological experts.

To our knowledge experiences and views of cancer survivors regarding smoking and smoking cessation has scarcely been investigated in qualitative studies to date. The decision to conduct qualitative research was made regarding the sensitive topic of persistent smoking in survivors after having been successfully treated for cancer. The qualitative research approach is well suited to behavioral and individual perspectives of complex concepts.^30^ An inductive approach was chosen because there is little evidence to date on the motivating factors for continuing or quitting smoking as well as on the strategies and barriers to smoking cessation in cancer survivors. The report for this study followed the 32-point checklist for reporting qualitative research (Consolidated criteria for reporting qualitative research).^31^

### Measures

A semi-standardized interview guide was developed (see Supplemental Materials S1) and used for its ability to be flexibly adapted by the interviewers to best capture areas of particular importance. More in-depth questions were asked and adapted based on survivors’ responses, focusing on their preferences and concerns.^32^ As this was a qualitative research approach, the goal was not to produce generalizable findings, but rather to understand individual perspectives, meanings, and experiences by taking sufficient time and tailoring the interviews to each participant. In the beginning of the interviews, screening questions such as age, gender, occupation were asked to obtain personal and medical data for a detailed sample description. Furthermore, all participating cancer survivors were screened for their smoking patterns and were asked about their used smoking products. Those who primarily smoked cigarettes were also screened for cigarette dependence using the Fagerström questionnaire.^33^ On this screening tool, 0-2 points indicate low physical dependence on tobacco, 3-4 points moderate dependence, 5-6 indicate high and 7-10 very high dependence on tobacco.

The guideline (see S1) contains all 14 main questions and six additional questions that were already phrased as in-depth questions. The questionnaire was developed specifically for this study and pilot-tested with three individuals prior to data collection (representing 50% of the final sample). Minor wording adjustments were made to improve clarity and flow.

### Participants and Recruitment

In this study, interviews with formerly or currently smoking cancer survivors were conducted. The inclusion criteria were a) being a current or former smoker and b) having been diagnosed with cancer and c) having successfully completed treatment at least 2 years ago, d) having no signs of relapse and e) being more than 18 years old. To be classified as a smoker, a person must have smoked at least 100 cigarettes in his or her lifetime. A former smoker was defined as someone who smoked at least 100 cigarettes in their lifetime, quit smoking at least 6 months ago and did not smoke a cigarette afterwards, while a current smoker is someone who continues to smoke either regularly or occasionally.^34^

Participants were excluded if they were not fluent in German or if they were in a physical or psychological condition that, in the judgment of the treating clinical team, did not allow for participation or was considered ethically inappropriate. To ensure heterogeneity, cancer survivors with any cancer type and treatment history were eligible to participate, provided they had sufficient fluency in German. Participants were recruited in person at oncological institutions in the metropolitan area of a major German city, as well as through various outpatient clinics across North Rhine-Westphalia.

Participants were recruited exclusively through snowball sampling, initiated via two main pathways. First, recruitment began within our own clinical setting. Initial participants were identified through existing professional and therapeutic networks at our institution. These individuals were then invited to share information about the study with others in their personal or treatment-related networks (eg, fellow patients, acquaintances), allowing the sample to expand gradually through peer referral.

Second, we used social media as an entry point for further participant recruitment. A digital flyer with study information was shared in relevant online communities and patient groups. Interested individuals could contact the research team directly. Consistent with the snowball sampling approach, those who participated were also encouraged to circulate the flyer within their own networks, thereby broadening recruitment through online peer-to-peer sharing.

Snowball sampling was chosen due to the limited public accessibility of the target group, cancer survivors. It was assumed that cancer survivors connected to the hospital’s cancer care network would be more aware of the relevance of the research topic and more open to sharing their experiences. Given the sensitive and personal nature of the topic, this sampling method also facilitated trust-based referrals, which supported participant engagement.

All interested participants received detailed information about the study, including eligibility criteria and data protection protocols. After providing informed consent, interviews were scheduled at the participant’s convenience.

Given the nature of snowball sampling, the total number of individuals who received the study invitation cannot be determined.^35^ Among those who were directly approached, the participation rate was approximately 50%. As this was a qualitative study, no formal power analysis was performed. The final sample consisted of six participants, and sample size was determined based on the principle of data saturation, which was achieved when no new themes emerged in the subsequent interviews.

### Data Collection

Most interviews were conducted by telephone, following a semi-structured interview guide. Interviews were audio-recorded and transcribed verbatim for subsequent analysis. All interviews took place between September 2021 and January 2022. Data collection was discontinued after six interviews because it became evident that relevant perspectives and experiences were captured, with no new emerging content dimension. The same trained research assistant conducted, audio recorded the interviews and took notes. For the interviews, participants were instructed to be alone in their room. The interview lengths varied between 30 and 50 minutes. The interview was only conducted once and there was no repetition of interviews. The transcript was kept private and not returned to the participants. Before the interviews, the interviewer briefly introduced herself and explained the objectives of the study.

### Ethics and Dissemination

The study was conducted in accordance with the Code of Ethics of the Declaration of Helsinki and approved by the Ethics Committee of the LPEK (Local Psychological Ethics Committee at the Center for Psychosocial Medicine Hamburg, Germany) (tracking number: LPEK-0324). All survivors received an information letter about the study via email or postally and signed a consent form before participation.

### Data Analysis

The audio recordings were verbatim transcribed using the transcription software “f4transcript” (Version 7) according to the transcription rules by Kuckartz.^36^ All personal data like names or places of residence were anonymized. Interviews were conducted and analyzed iteratively, with emerging themes reviewed after each transcript. By the fifth and sixth interviews, no new themes or codes were identified. This was discussed within the research team, and consensus was reached that thematic saturation had been achieved. To enhance the credibility of the assessment, multiple researchers were involved in this process, as well as in the process of coding and theme development. Data was further analyzed with the software program Qualitative Content Analysis (QCAmaps) using an inductive approach.^37^ The process model according to ‘Mayring’ was used.^38^

After defining the research questions, the selection criteria (all text passages) and the abstraction level (medium) were established. In addition, the units of analysis were determined as follows: sentence parts were chosen to be the smallest allowed text components to be categorized, the entire interview was chosen as the context unit, and the entire text material was chosen as the unit of analysis. After coding approximately 50 percent of the material, a formative reliability check was conducted. During this process, two researchers independently analyzed the interviews to determine whether they reached similar results. Interrater reliability was calculated using Cohen’s Kappa, with a value of .80 indicating strong reliability and substantial agreement.^38^ Any discrepancies were resolved through further discussion. When working through the material line by line, a category system with main categories (marked with CX), subcategories (marked with CX.X) and further sub-subcategories (marked with CX.X.X.) were developed, as described by Mayring. Through a rule-guided reduction, a summary of the data material was created.^38^ The following quality criteria for qualitative research were met: procedural documentation, rule conformity, transparency, empirical anchoring, and reliability testing by re-categorization at a different time by the same person and also a different person.^39^ Participants were not involved in the analysis process nor did they provide feedback on the findings.

## Results

### Sample

Six cancer survivors (50% female) with different cancer types (breast tumor, GIST tumor, skin cancer, lung cancer, and urinary bladder cancer) participated in this study, ranging in age from 34 to 81 years. Four of the six participants were married. Three participants were retired, one was a housewife, and two were employed. All had undergone cancer treatments including surgery, chemotherapy and hormone therapies. After treatment participants had regularly conducted follow-up care.

All patients reported being at least four years after the end of therapy for their specific cancer and free of recurrences. Of the six interviewees, three survivors were still smoking and had never stopped, the other three had stopped smoking after cancer diagnosis (n = 2) or before (n = 1). Of the current smokers, two smoked cigarettes regularly and one consumed a heat-not burn product called “I Quit Ordinary Smoking”. On the dependence scale (Fagerström-Test), all smokers showed strong dependence on tobacco with scores of 5 and above.^33^

### Results of the Qualitive Analysis

**Figure 1.**
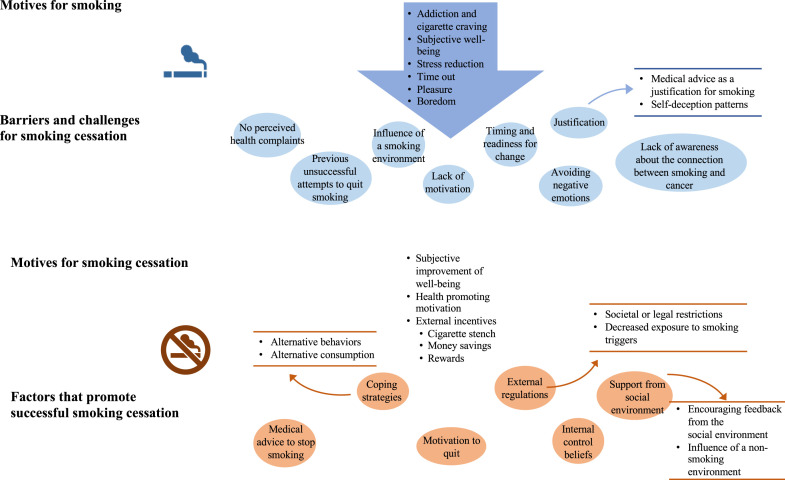
Visualization of the Qualitative Results

The results will be explained based on the inductive main categories, subcategories and sub-subcategories built from the material. All identified categories are visualized in Figure 1. Four main categories emerged with differing amounts of sub- and sub-subcategories:

• Motives for smoking (C1); five subcategories (C1.1-C1.5)
• Motives for smoking cessation (C2), three subcategories (C2.1-C2.3), four sub-subcategories (C2.3.1 - C2.3.3)
• Opportunities and facilitators of successful smoking cessation (C3) six subcategories (C3.1-3.6) and six sub-subcategories (C3.4.1- C3.4.2); (C3.5.1- C3.5.2); (C3.6.1-C3.6.2).
• Barriers and challenges for smoking cessation (C4) eight subcategories (C4.1- C4.8) and two sub-subcategories (C4.1.1- C4.1.1)

All categories are further illustrated in Figure 1.

All derived categories from the data are illustrated in Figure 1. The image was divided into two sections. The upper area represents the motives for smoking (C1) and the barriers and challenges for quitting smoking (C4). The lower part of the image summarizes the motives for quitting smoking (C2) as well as the opportunities and facilitators of successful cessation.

#### C1: Motives for Smoking

The main category C1 emerged from the data and can be used to answer the first research question: “Which factors motivate cancer survivors to continue smoking?”. Various motives for smoking emerged in the data that explain why survivors continue to smoke after diagnosis. One subcategory represents Craving (C1.1), ie, the urgent desire to smoke due to tobacco addiction, and the relief they feel when this craving is satisfied.

“(...) the craving for smoking is satisfied and I no longer feel that pressure (...)” (person 3)

Another subcategory is stress reduction (C1.2). Smoking is described to provide relaxation and stress relief, especially when experiencing negative feelings in unpleasant situations. The category time out (C1.3) describes how individuals use smoking as a break from routines of everyday life.

“I often took it as time-out (…), stepping out for a smoke meant I was taking a break - five minutes of peace (...)” (person 2)

Further motives for smoking are gain of pleasure (C1.4) and boredom (C1.5).

#### C2: Motives for Smoking Cessation

The main category C2 summarizes motives for smoking cessation and can be assigned to the second research question: “Which factors motivate cancer survivors to cease smoking?” This category resumes advantages and positive aspects that have motivated cancer survivors to quit smoking.

The subcategory subjective improvement of well-being (C2.1) summarizes descriptions about an increased quality of life and performing better in daily life without smoking.

“I think [after smoking cessation] one feels different or free or not so bad anymore.” (person 6)

Correspondingly, the health promoting motivation (C2.2) subcategory recaps reports from survivors about the desire to improve their health by quitting smoking.

“The main reason to stop smoking was my health condition.” (person 4)

Another aspect is summarized in the subcategory external incentives (C2.3), which includes external aspects and consequences that can promote smoking cessation. Sub-subcategory of C2.3 include getting rid of the typical smoking odor associated with smokers (cigarette stench) (C2.3.1), of having more money because one does not have to buy cigarettes (money savings) (C2.3.2), as well as other incentives such as health insurance bonus program rewards (C2.3.3).

“Motivation isn’t just about having a goal; it’s also an extreme financial matter. There are bonus programs from health insurance companies (...) and you get a nice reward because we save on hospital costs - probably enough for a wellness weekend or something genuinely enjoyable.” (person 2)

#### C3: Opportunities and Facilitators of Successful Smoking Cessation

The next major category C3, including six subcategories, was later assigned to the third research question:“Which strategies are used by cancer survivors who have tried or succeeded in quitting smoking?”. It includes aspects that have positively influenced or are expected to support smoking cessation. The first subcategory is about internal control belief (C3.1). To successfully quit smoking, cancer survivors reported that successful smoking cessation depends heavily on one’s own personality and control beliefs.

“(...) it really depends on your own personality. How strong am I?” (person 1)

Another subcategory is medical advice to stop smoking (C3.2): cancer survivors who quit smoking and maintained their abstinence reported being significantly influenced by medical recommendations. They noticed that their physicians’ encouragement to stop smoking played a crucial role in their decision to quit.

“Yes, because the doctors told me that with lung cancer, the worst thing you can do is smoke. Otherwise, I wouldn't have quit. (…) Out of necessity, I pulled myself together and stopped.” (person 5)

“(...) I was supposed to have my reconstruction surgery (…), and the message was: ‘If you don’t stop smoking (…) beforehand, we won’t do the surgery.’ Well, I stopped.” (person 2)

Motivation to quit (C3.3) represents another subcategory on facilitators to smoking cessation.

“If you want to quit smoking, your motivation has to be strong enough to make it happen.” (person 3)

The subcategory external regulations (C3.4) summarizes aspects that are restricting smoking and thereby may lead to smoking cessation. Societal or legal restrictions (C3.4.1) can be experienced when regulations influence smoking behavior (eg, smoking bans in public spaces).

“Since smoking is no longer allowed in pubs, I smoke much less, and I really appreciate that.” (person 2)

Regulations and restrictions also contribute to decreased exposure to smoking triggers (C3.4.2). Cancer survivors report that avoiding certain stimuli associated with smoking, such as limiting alcohol consumption, greatly aids their efforts to remain smoke-free. Another subcategory describes support from the social environment (C3.5) and was divided into encouraging feedback from the social environment (C3.5.1), eg, positive reactions on their quit attempts, and the positive influence of a non-smoking environment (C3.5.2), eg, being surrounded by non-smokers.

Another subcategory was created to summarize coping strategies for cigarette cravings (C3.6). Cancer survivors reported engaging in alternative behaviors (C3.6.1), eg. cleaning the apartment to resist the urge to smoke, and alternative consumption (C3.6.2), eg. eating sweets as a strategy against craving.

“Then you might eat some sweets or find something to occupy yourself with. You know you are thinking about it, but you just try to do something where you don't have to focus on it.” (person 6)

#### C4: Barriers and Challenges for Smoking Cessation

Another main category summarized barriers and challenges (C4) and can be used to answer the fourth research question, “What obstacles are encountered in the attempt to quit smoking?”. This category describes reported aspects that make it difficult for cancer survivors to quit smoking. The subcategory justification of continued smoking (C4.1) describes various ways cancer survivors rationalize continued smoking post-diagnosis, despite not being proud of it. The sub-subcategory medical advice as justification for delayed smoking cessation (C4.1.1) includes survivors’ remarks about feedback from oncological staff, who indicated that immediate cessation of smoking was not essential.

“Then the doctor told me, ‘Okay, don’t think about it for now, and please try to reduce after chemo when everything is back to normal.’” (person 2)

Another part of justification was summarized as self-deception patterns (C4.1.2). Cancer survivors described how they cheated on themselves by engaging in behaviors that didn’t truly contribute to their goal of quitting smoking but offered a temporary relief.

“Cigarettes are very expensive, after all, but I just snap half off, take three puffs, and that’s it. Then I’m satisfied with just half a cigarette. I throw the other half away.” (person 6)

Another subcategory, lack of awareness regarding the connection between smoking and cancer (C4.2), addresses survivors’ reports of not recognizing the link between smoking and their cancer diagnosis. In addition, cancer survivors reported that they continued to smoke to avoid negative emotions associated with smoking cessation (C4.3). They report experiencing unhappiness and extreme stress after attempting to quit, feelings they feared would never go away, making it difficult for them to quit.

“It puts you under so much stress and you feel like it's not going to stop for the rest of your life. (...) I find that feeling a lot worse than those few days in the beginning [after cessation].” (person 2)

In addition, the timing for cessation and readiness for change (C4.4) is considered by smoking cancer patients. Some feel like the period immediately following their diagnosis is not the right time to quit, as they are dealing with numerous health issues related to their disease and treatment and would not be able to take up a second challenge. They feel that another moment might be more suitable to pursue smoking cessation.

“You are in the hospital, lying there flat, (…) and it makes you really nervous. (…) If you stop smoking now, you're really going to go crazy.” (person 3)

Another subcategory summarizes the influence of a smoking environment (C4.5), as a smoking peer group is often considered a barrier to quitting.

According to the cancer survivors, previous unsuccessful attempts to quit smoking (C4.6) may also operate as a barrier for further smoking cessation attempts.

“I tried to quit and I struggled day by day. After three or four months, I started smoking again. I attempted this several times.” (person 3)

Another barrier is the lack of motivation (C4.7) to quit and having only few physical or psychological limitations or consequences due to current smoking behavior (no perceived health complaints C4.8).

“And at some point, I said: ‘Now I don’t care.’ (…) Why should I deal with this issue at 70 and try to quit smoking? I lack the motivation. Whether I live three years longer or die three years sooner doesn’t matter to me. It’s all coming to an end anyway (…) that’s not a reason for me to say, ‘I’m going to put myself through this torture and quit smoking.’” (person 3)

“I don’t feel any discomfort due to smoking, (…) I don’t have any significant health problems that I can feel, which is why I can’t seem to get it together.” (person 3)

All results are presented in tabular form with example citations in S2.

## Discussion

The interviews with cancer survivors revealed several aspects that can motivate smoking cessation after a cancer diagnosis, but also a multitude of inner conflicts and barriers, which make it difficult for cancer survivors to quit smoking. Furthermore, a variety of different aspects that contribute to continued smoking were reported.

According to the cancer survivors interviewed for this study, one primary motive for continued smoking was experiencing symptoms of cigarette craving (C1.1). Participants described only being able to endure certain periods of time without smoking and feeling a significant relief when smoking a cigarette. Nicotine craving is estimated to be one of the most common withdrawal symptoms in the general smoking population, making it especially difficult to abstain from smoking.^40^ Likewise, craving and withdrawal symptoms were mentioned as important barriers to cessation in other research on smoking cessation in patients with cancer.^41^ While evidence-based cessation programs include the use of medication to reduce these symptoms, other research revealed negative beliefs about these medications among cancer survivors with concerns about safety and possible side effects.^42^ For instance, patients were unsure about possible interactions with their cancer treatment.^42^ Cancer survivors in this study also reported that smoking can work as a coping strategy to reduce treatment related stress and improve the subjective well-being in certain situations eg. feeling calmer and more relaxed (C1.2). One of the most frequently cited motives for smoking was the perceived resulting reduction of stress. However, the physiological effects of nicotine also lead to an increased heart rate, an important part of stress response and arousal.^43,44^ Hoover et al. highlighted the importance of implementing stress reducing programs into cancer care and cessation support, such as general wellness programs or stress management courses.^45^

Other motives for smoking mentioned during the interviews were pleasure (C1.4), boredom (C1.5) and having a time-out (C1.3). Cancer survivors reported to use smoking both as means of enjoyment and as an occupation when being bored, which is similar to findings in the general smoking population.^46^ However, distraction and enjoyment may have a completely different meaning for cancer survivors: after cancer treatment, many do not feel psychologically well^17^ and exhibit poorer social functioning.^17,47^

For participants, motives for smoking cessation (C2) could be improved well-being (C2.1) and health (C2.2). Likewise, Berg et al. reported that positive health outcomes can be an important motivator for smoking cessation in patients with cancer.^41^ Research could also show that cancer survivors who quit smoking experienced a greater reduction of depressive symptoms also leading to better well-being compared to cancer survivors who continued smoking.^4,5,41^ Furthermore, external incentives (C2.3) like getting rid of the cigarette stench (C2.3.1) or saving money (C2.3.2) play an important role in facilitating smoking cessation. Data on smoking cessation treatments for cancer survivors is growing, with recent studies highlighting the effectiveness of evidence-based behavioral and pharmacological interventions, that can also improve long-term outcomes.^12,41,42,48^

Our findings generated several factors that facilitate success of a smoking cessation. One factor retrieved was internal control belief (C3.1). Cancer survivors reported that successfully quitting smoking would depend on one’s own personality, will or conviction, and will require strong discipline and the ability to endure the pressure of craving. Also, receiving medical advice (C3.2) was listed as an important factor to promote successful smoking cessation. Cancer survivors reported that being asked directly by their oncological professional to quit smoking had led them to stop smoking and remain abstinent. Similarly, other research highlighted the importance of a doctor’s advice in facilitating smoking cessation in cancer survivors.^41^ Noteworthy, studies also criticized the lack of medical advice or screening regarding smoking status by oncology professionals.^8,49^ In a large population-based study it was observed that only half of the patients with cancer received advice to quit smoking.^50^ Our findings reveal a notable inconsistency in the smoking cessation messages communicated by healthcare providers. While some participants reported receiving clear and motivating advice to quit smoking, others described being advised to delay cessation or receiving no guidance at all. This variation may reflect a lack of standardized institutional protocols, differing personal attitudes among clinicians, or uncertainty regarding the timing of cessation in relation to treatment. Given the established benefits of smoking cessation for cancer patients, particularly in improving treatment outcomes and quality of life, such discrepancies are concerning. Leading cancer care and health authorities consistently emphasize the importance of integrating evidence-based tobacco cessation support into cancer care, and advocate for universally applicable policies and practice guidelines.^9-11^ Consistent, evidence-based messaging from healthcare providers is crucial, as medical advice often plays a central role in motivating behavioral change. Addressing this issue, for example by including standardized protocols and regimes regarding cessation advice into cancer care could enhance the support patients receive and reduce confusion or ambivalence about quitting. Furthermore, the results highlight the moment after a cancer diagnosis to be used by oncology professionals to educate and motivate survivors to stop smoking. Unfortunately, the “teachable moment” is still underused in the clinical reality of oncology care.^51^ One review that examined smoking cessation appeals by physicians in the oncology setting reported that physicians typically emphasized the potential financial savings from quitting smoking.^52^ Saving money due to less expenses for cigarettes was mentioned as a motivator for smoking cessation by participants in this study. Simultaneously, other research reported that many cancer patients struggle financially.^53^ It seems like this motivator for quitting smoking may have already reached the awareness of oncology treatment providers.

Other mentioned factors to support smoking cessation were external regulations, such as societal or legal restrictions (C3.4), which permitted smoking only in certain situations and public spaces and therefore reduced the number of cigarettes smoked. The same has been widely evaluated in studies of the general smoking population: restricting smoking at work and at home was associated with increased quit attempts, decreased relapse rates, and a lower amount of smoking products per day.^54^ A systematic review from 2010, including several countries, such as the U.S., Canada, Australia, Germany and France, showed that smoke-free policies were associated with an average decrease of 3.4% in the prevalence of smoking.^55^ Cancer survivors also noted that stimulus control, such as staying away from situations that promote smoking, was helpful in quitting smoking. For example, when a participant abstained from alcohol or parties, they could decrease their cigarette consumption because these situations were associated with smoking. Particularly alcohol consumption seems to play an important role in smoking and cessation. Britton et al. (2021) were able to show that in a smoking population of Latin Americans, smoking cessation worked less effectively for individuals who simultaneously consumed alcohol.^56^ In other qualitative studies, a smoke-free environment was recognized as an essential part of successful smoking cessation,^57^ while being surrounded by smokers was recognized as an important barrier.^12,41^ Likewise, the influence of the social environment (C3.5) was recognized by cancer survivors, which can either support or hinder successful smoking cessation (C3, C4.5). Positive feedback of quit attempts from friends or relatives was perceived as highly important and encouraged cancer survivors to quit for good. This aligns with other qualitative research indicating that lower perceived support from the significant other or the social environment predicted continued smoking after a cancer diagnosis.^41^

Finally, individual coping strategies for cigarette craving (C3.6) are important components of successful cessation. One strategy might be discovering alternative activities (C3.6.1), such as going outside or watching television. But also, the consumption of other addictive substances such as eating sweets can serve as an alternative to smoking. Interestingly, none of the participants reported having used evidence-based smoking cessation therapies, such as pharmacological treatments (eg, bupropion, varenicline), with or without counseling to successfully quit smoking.^58^ This is a noteworthy finding, given that such evidence-based interventions could potentially address several of the concerns expressed by participants regarding the cessation process, particularly fears around cravings and doubts about the ability to quit successfully. It remains unclear whether these treatment options were unknown to participants, not offered by healthcare providers, or actively declined due to negative beliefs about these medications, skepticism, or misinformation. The participants who referred to pharmacological options appeared uncertain about potential side effects or possible interactions with their cancer treatment. This, combined with a general lack of knowledge about the efficacy and safety of these interventions, suggests that participants were both underinformed and insecure. Simultaneously, many described strong fears related to cravings and withdrawal symptoms – issues that could be effectively mitigated through appropriate pharmacotherapy or behavioral support. The findings generally underscore the need for improved patient education, clear communication from healthcare providers, and proactive cessation counseling in oncology care. Addressing informational gaps about available cessation treatment options can improve acceptance and uptake of cessation aids but also reduce anxiety and enhance confidence in the quitting process.

One of the most often quoted challenges for quitting (C4) was the fear of handling negative emotions (C4.3), which is comparable to other research highlighting that experiencing stress after cessation was a significant barrier to quitting.^41^ During cessation, feelings of stress, anxiety or insecurity can occur. In addition, previous experiences with unsuccessful attempts (C4.6) were reported by the cancer patients. With failed quit attempts, motivation to try smoking cessation may decrease. In a study of the general smoking population, smokers with unsuccessful quit attempts showed higher levels of distress compared to other smokers or former smokers (van der Deen et al, 2011). However, previous quit attempts may also be predictors of further attempts to quit smoking.^59^ A lack of motivation to quit (C4.7) was further discussed as another barrier. Often cancer survivors do not express the desire to stop smoking. Especially after former failed quit attempts, smokers expressed a rather hopeless attitude. Literature has shown that motivation to quit smoking predicts the success of subsequent smoking cessation.^60^

In this study, smoking cancer survivors reported not having subjective health complaints (C4.8) from smoking, although smoking has been shown to have negative physical and psychological effects, especially among cancer survivors.^4^ This indicates that patients were generally not understanding or not being aware of the connection between smoking and one’s cancer diagnosis (C4.2). Oftentimes cancer survivors find it difficult to accept that smoking may have influenced the development of their tumor and downplay its impact compared to other factors such as environmental aspects (Faller et al, 1995). Likewise, in other research cancer survivors knew only very little about the connection between smoking and cancer, the health consequences and adverse effects of continued smoking.^41,45^ On the one hand this may be due to poor patient education, on the other hand this may also be a coping mechanism. Lung cancer patients who made causal attributions suffered from high levels of negative emotions, were more depressed and had less hope compared to other cancer patients. It is important to avoid blaming cancer survivors who smoke or overemphasizing the detrimental health effects of smoking. Instead, the focus should be places on the substantial benefits of cessation.^45,61^ Positive health outcomes were an important motivator of smoking cessation in other research.^41,45^ This also corresponds to another subcategory, the justifications (C4.1) smokers bring up of continued smoking. Cancer survivors reported that they acted upon medical advice and spoke about delaying to finally quit smoking or only smoking half of their cigarettes to make them feel better. These statements can be interpreted as cognitive distortions and trivializations of continued smoking and might be defense mechanism used to distort perceptions of one’s behavior. The use of a variety of cognitive mechanisms to justify smoking behavior may help smokers to reduce negative feelings associated with inner conflicts.^62^ This aligns with Festinger’s (1957) theory of cognitive dissonance.^63^

Another controversial topic in the interviews was the question of the right timepoint to be educated (C4.4) about the consequences of continued smoking. The idea of quitting smoking right after the diagnosis triggered anxieties along with the psychological burden of the oncologic disease, treatments, and hospitalizations and was perceived as the wrong time to be educated and motivated to quit smoking (C4.4) by cancer survivors. A study by Streck and colleagues (2021) confirmed that cancer survivors are exposed to high levels of psychological distress after diagnosis.^7^ Then again, the time after diagnosis is referred to as a “teachable moment”^64,65^ and experts recommend using this opportunity for change especially in high-risk patients such as cancer patients. The “teachable moment“ increases awareness of the negative consequences of smoking and thus raises motivation to quit smoking.^64,65^ Interestingly, only those who had already successfully quit smoking recognized the “teachable moment” as an important possibility to change dysfunctional health behavior.

All in all, our findings provide important insights into reasons for continued smoking in cancer patients as well as their perceptions about smoking cessation and the process of quitting. The results offer explicit and practical information about which aspects are perceived as supportive or discouraging for successful quitting, and how and when clinicians can effectively assist patients - while using evidence-based strategies, as recommended in previous studies.^12,41^

Education should be provided on how smoking affects cancer treatment, course of the disease and psychological well-being. In addition, coping strategies are needed for both stressful moments and cigarette cravings. Alternative activities must be explored, which provide pleasure or serve as a time-out. Interventions could include the involvement of relatives, who should learn how to support cancer survivors in quitting smoking.^66^ Motivational therapy should be used to build or increase motivation and achieve long-term smoking cessation.^45,35^

Internal dissonances regarding cessation could be resolved by cognitive behavioral therapy interventions including motivational interviewing.^67^ This approach may be particularly helpful for persistent smokers who display a lack of willingness to change.

Furthermore, lack of awareness or misinterpretation of physical symptoms may indicate a lack of self-care, that could be addressed in psychosocial interventions to enhance self-esteem.^68^

The results also highlighted the importance of the doctor-patient relationship regarding medical education about the consequences of continued smoking as well as strong advice to quit. The “teachable moment” proves to be a central tool during oncology care. Survivors who continued smoking after their diagnosis missed the opportunity of the “teachable moment”^51^ in our study, whereas those who actually managed to quit successfully were able to take advantage of this “teachable moment”. It is both a combination of the cancer survivors’ acceptance and the physicians’ preparedness to recommend smoking cessation to cancer survivors. As the hospitalization can be a time-out from the addictive environment it may represent a good timepoint for cessation. Integrating a smoking cessation intervention into the hospital setting should be the goal to help cancer survivors quit smoking, remain abstinent and achieve better health outcomes.

### Limitations

The search for motivated eligible participants appeared to be difficult. In most cases, the initial contact was unsuccessful and so ultimately, the snowball sampling was found to be a useful method.^35^ However, it is possible that this limits the representativeness of the sample. Another important limitation of this study is the small sample size and the absence of a formal power analysis, which, although common in qualitative designs, may limit the transferability of findings. Furthermore, the time- and resource-intensive interviewing strategy and qualitative research approach justifies a smaller sample size. When no new insights emerged after a certain point, it was not to be expected that a larger number of interviews would add to the theoretical insights. The focus of this study was on depth of analysis and the understanding of individual perspectives. The large diversity in age and cancer type within the sample provided various perspectives on the issue, including different smoking behaviors and durations of smoking over a lifetime. Including participants from other cancer types or underrepresented age groups could have yielded further insights. This could be an interesting approach to future research in this area. Notably, the use of a snowball sampling method inherits the possibility of selection bias, as participants may refer individuals with similar backgrounds or perspectives. This may have influenced the range of perspectives included in the study. To address this, we made efforts to guide the referral process by encouraging diversity in age, gender, and cancer types where possible. Future research would benefit from combining snowball sampling with other recruitment strategies to mitigate such bias and enhance sample heterogeneity.

It is important to note that the interviews included one former smoker who quit smoking independently of the cancer disease. It was decided not to exclude the person, as this person was still able to give insights into past smoking patterns, experiences with smoking cessation attempts, and the challenges of remaining smoke-free. Although the questionnaire was pilot-tested to ensure comprehensibility, its lack of formal validation represents a methodological limitation.

While conducting the interviews, the researcher further noticed that some cancer survivors had difficulty verbalizing their own thoughts. This may be related to their oncological disease or psychological complaints, such as the often-experienced fatigue syndrome.^69^ Then again, smoking may also be an extremely sensitive topic to talk about. The researcher therefore behaved in an interactive and attentive manner, adapting to the survivors to create a trustful atmosphere. This behavior could have influenced the behavior of cancer survivors as well as the content of their narrative.

## Conclusion

The results highlight significant deficits in smoking cessation interventions during both the treatment and aftercare of cancer survivors. The inconsistency in smoking cessation advice reported by participants underscores the need for more standardized, evidence-based communication by healthcare providers in oncology settings. Clear and supportive messaging may play a decisive role in patients’ motivation and ability to quit smoking, particularly during critical periods of their care. Given the vulnerability of cancer survivors to the detrimental effects of smoking, it is of great importance to improve cessation rates, thereby enhancing both their physiological and psychological well-being.

The findings suggest that existing smoking cessation interventions should be further developed to address the specific needs of this population. Policies must be tailored to these needs and integrated into clinical routines. Recognizing the “teachable moment” following a cancer diagnosis, alongside consistent medical advice, can play an important role in facilitating smoking cessation. Oncology professionals have a key responsibility to ensure that these interventions are effectively implemented, ultimately improving outcomes for cancer survivors.

## Supplemental Material

Supplemental Material - Formerly Smoking and Currently Smoking Cancer Survivors’ View on Smoking Cessation – A Qualitative Study Supplemental Material for Formerly Smoking and Currently Smoking Cancer Survivors’ View on Smoking Cessation – A Qualitative Study by Frederike Bokemeyer, Johanna Springorum, Lisa Lebherz, Carsten Bokemeyer, Holger Schulz, Kathleen Gali, Christiane Bleich, Paulina Kiefer, Sven Püffel, and Janina Freitag in Tobacco Use Insights.

## Data Availability

This publication contains all relevant data. Detailed data will be made available upon demand, eg, for systematic reviews or meta-analyses.*
