# Supplementary material for: Formerly Smoking and Currently Smoking Cancer Survivors’ View on Smoking Cessation – A Qualitative Study
Source: Tob Use Insights. 2025 Oct 9;18:1179173X251355531. doi: 10.1177/1179173X251355531 (PMC12511738; doi:10.1177/1179173X251355531)
Supplement: Supplemental Material - Formerly Smoking and Currently Smoking Cancer Survivors’ View on Smoking Cessation – A Qualitative Study [file sj-pdf-1-tui-10.1177_1179173X251355531.pdf]

S1 Data Interview guideline

	Original Questions (German)	Suggested follow-up Questions (German)	Translated Questions (English)	Suggested follow-up Questions (English)
1	Welche Bedeutung hat das Rauchen für Sie?	1.1 Wie fühlen Sie sich, wenn Sie rauchen?<br>1.2 Was bewirkt das Rauchen bei Ihnen?	What does smoking mean to you?	1.1 How do you feel when you smoke?<br>1.2 What effect does smoking have on you?
2	In dem Moment, als Sie von Ihrer Krebsdiagnose erfahren haben, welche Gedanken sind Ihnen bezüglich Ihres Rauchverhaltens durch den Kopf gegangen?	2.1 Hat sich die Bedeutung des Rauchens für Sie durch die Krebsdiagnose geändert?	When you learned about your cancer diagnosis, what thoughts did you have regarding your smoking habits?	2.1 Has the significance of smoking changed for you since your cancer diagnosis?
3	Was würde es für Sie bedeuten weniger zu rauchen?		What would reducing smoking mean to you?	
4	Wofür brauchen Sie das Rauchen?		Why do you smoke?	
5	Welche positiven und negativen Konsequenzen würden Sie bei der eigenen Raucherentwöhnung für Sie erwarten?		What positive and negative effects do you expect if you quit smoking?	
6	Wie sahen bisherige Versuche der Raucherentwöhnung bei Ihnen aus?	6.1 Was hat Ihnen am meisten und am wenigsten geholfen?	What did previous attempts to quit smoking look like?	6.1 What helped you the most and least during past quit attempts?
7	Wie haben Ihre behandelnden Ärzte oder Therapeuten Ihren Rauchstatus angesprochen?		How did your healthcare providers address your smoking status?	

8	Wie wurden Sie über die Folgen fortgesetzten Rauchens nach der Krebsdiagnose aufgeklärt?	Wie haben Sie eine Aufklärung über die Folgen des Weiterauchens nach einer Krebsdiagnose erlebt?	How were you informed about the consequences of continued smoking after your cancer diagnosis?	8.1 What was it like for you to learn about the consequences of continued smoking after your cancer diagnosis?
9	Würden Sie eine Unterstützungsmöglichkeit nutzen, um eine Raucherentwöhnung durchzuführen?		Would you consider participating in support options for quitting smoking?	
10	Was würde Sie anregen eine Unterstützungsmöglichkeit zu nutzen?	Was motiviert Sie mit dem Rauchen aufzuhören	What would encourage you to use smoking cessation support?	10.1 What motivates you to quit smoking?
11	Wen Sie sich ein Leben ohne das Rauchen vorstellen, gibt es dann auch etwas das Ihnen schwer fällt bzw. etwas das Ihnen fehlen würde?		If you imagine a life without smoking, is there something that would be difficult for you or something that you would miss?	
12	Welche Hindernisse sehen Sie bei der Nutzung einer Unterstützungsmöglichkeit?		What do you see as potential barriers to using smoking cessation support?	
13	Welche Rolle könnte das soziale Umfeld bei einer Unterstützungsmöglichkeit für Sie bedeuten?		What role might your social environment play in your use of smoking cessation support?	
14	Wenn alles möglich wäre, was würden Sie sich für ein		If anything were possible,	

	rauchfreies Leben wünschen ?		what would you wish for in a life without smoking?	
